# Ginseng and Ginkgo Biloba Effects on Cognition as Modulated by Cardiovascular Reactivity: A Randomised Trial

**DOI:** 10.1371/journal.pone.0150447

**Published:** 2016-03-03

**Authors:** Derek Ong Lai Teik, Xiao Shiang Lee, Chu Jian Lim, Chia Mei Low, Mariyam Muslima, Luca Aquili

**Affiliations:** 1 Department of Psychology, Sunway University, Bandar Sunway, Malaysia; 2 Department of Marketing, Sunway University, Bandar Sunway, Malaysia; Kurume University School of Medicine, JAPAN

## Abstract

**Background:**

There is some evidence to suggest that ginseng and Ginkgo biloba can improve cognitive performance, however, very little is known about the mechanisms associated with such improvement. Here, we tested whether cardiovascular reactivity to a task is associated with cognitive improvement.

**Methodology/Principal findings:**

Using a double-blind, placebo controlled, crossover design, participants (*N* = 24) received two doses of Panax Ginseng (500, 1000 mg) or Ginkgo Biloba (120, 240 mg) (*N* = 24), and underwent a series of cognitive tests while systolic, diastolic, and heart rate readings were taken. Ginkgo Biloba improved aspects of executive functioning (Stroop and Berg tasks) in females but not in males. Ginseng had no effect on cognition. Ginkgo biloba in females reversed the initial (i.e. placebo) increase in cardiovascular reactivity (systolic and diastolic readings increased compared to baseline) to cognitive tasks. This effect (reversal) was most notable after those tasks (Stroop and Iowa) that elicited the greatest cardiovascular reactivity during placebo. In males, although ginkgo also decreased cardiovascular readings, it did so from an initial (placebo) blunted response (i.e. decrease or no change from baseline) to cognitive tasks. Ginseng, on the contrary, increased cardiovascular readings compared to placebo.

**Conclusions/Significance:**

These results suggest that cardiovascular reactivity may be a mechanism by which ginkgo but not ginseng, in females is associated with certain forms of cognitive improvement.

**Trial Registration:**

ClinicalTrials.gov NCT02386852

## Introduction

Ginseng and Ginkgo Biloba are two of the most widely consumed herbal nutritional products in the world. Although historically these two products have been used in the treatment and prevention of a variety of diseases, particularly in traditional Chinese medicine, the last fifteen years have seen a substantial increase in their use as cognitive enhancers. The evidence for a beneficial effect of ginseng on cognition is mixed. A review of studies that have adopted stringent clinical trial criteria such as double-blinding, randomization, placebo-controlled, and crossover designs show contrasting results in terms of ginseng effects on working memory, attention, concentration, speed of processing and reaction time [1–5]. Additionally, a research group found an effect of ginseng on concentration but not on speed of processing in one study [6], and reported opposite outcomes in a follow-up investigation [7]. Such variability in findings is not entirely surprising, particularly in light of the large methodological differences across studies. Variables such as ginseng dosage, ginseng composition (i.e. type of ginsenosides present in the extract), characteristics of participants (i.e. healthy adults versus a clinical population), cognitive domain assessed, time point of cognitive assessment, washout periods, trial design (i.e. crossover, double-blind, placebo controlled, acute/chronic effects), and practice effects in crossover studies, render the task of evaluating the effectiveness of ginseng as a cognitive enhancer very difficult. This point also applies to research in any other cognitive compound.

The evidence for the effectiveness of ginkgo biloba as a cognitive enhancer is also somewhat mixed, particularly in the area of memory with some positive [8–13] and negative findings [14–19]. There appears to be a trend for a beneficial effect of ginkgo on various measures of attention [5, 20–22], although some negative findings have also been reported [10, 15].

Despite the vast array of studies carried out on the effects of ginseng/ginkgo biloba administration on cognitive performance, few attempts have been made to understand their physiological correlates. Some studies, for example, have attributed the beneficial effects of ginseng administration on working memory (e.g. serial threes) to reductions in blood glucose levels [6, 23]. Others [24], have shown that ginseng and ginkgo change cerebroelectrical activity by shortening the latencies of auditory-evoked potentials, and reducing theta and beta activity. Thus, a particular aim of this study was to understand whether the beneficial effects of ginseng/ginkgo may be related to another physiological measure, namely, stress reduction. Administration of ginseng (ginsenosides Rb1), in fact, has been shown to reduce catecholamine secretion (a marker of stress), in rats [25, 26]. Ginkgo has also been shown to modulate cortisol release and blood pressure in a stress model test [27]. Studies that have looked at the relationship between stress and cognitive performance demonstrate that stress has a detrimental effect on measures of cognitive flexibility in healthy participants [28, 29] and individuals with posttraumatic stress disorder show deficits in attentional set-shifting as modulated by dysfunctional dorsolateral prefrontal cortex and insula activity [30]. In the animal literature, stress by cold exposure impairs reversal learning and attentional set-shifting, and this deficit can be reversed by the administration of monoaminergic antidepressants [31]. If stress impairs measures of executive functioning such as cognitive flexibility, interventions that reduce stress should have the opposite effect. In fact, meditation and mindfulness practices have been shown to improve performance on response inhibition/cognitive flexibility (Stroop task)[32–34] and decision-making (Iowa Gambling task) [35].

Thus, as a specific proxy for stress, this study aimed to understand whether ginseng and ginkgo may affect the cardiovascular system and whether this in turn was a mediator of cognitive performance. This is in part because, for example, regions of the brain such as the prefrontal cortex, known to be involved in cognitive functioning, also appear to exert control over cardiac activity via a prefrontal cortex, amygdala, medulla pathway [36, 37]. The cardiovascular system, in turn, appears to modulate cognitive function. Heart rate reactivity, for example, predicts executive functioning, intelligence scores and performance on the Stroop task and Tower of London [38–41] and stress blunts the cardiovascular response which is predictive of depression (heart rate reactivity) and poor reversal learning performance (blood pressure reactivity) [42–44], although some studies suggest that enhanced and not blunted stress-induced blood pressure is associated with poor performance on memory and executive functioning [45, 46]. Blood pressure reactivity is also modulated by cognitive demand, as mental effort increases systolic and diastolic readings [47, 48]. Additionally, tasks with higher cognitive demand affect self-reported measures of stress [49], increase cortisol concentrations [50] and are reflected by changes in skin conductance and pupillary dilation [51, 52].

In the present study, blood pressure and heart rate measurements were taken after the completion of cognitive tests that measured different cognitive domains (simple attention/vigilance, impulsivity/behavioural flexibility, and complex executive functioning). It was hypothesised that ginseng and ginkgo biloba would selectively improve performance on these tasks that, during placebo, elicit the greatest increases in cardiovascular reactivity, and reflected in decreases in blood pressure reactivity (BPR) and heart rate reactivity (HRR) via its stress relieving actions. It was also predicted that ginseng and ginkgo may or may not improve performance on measures of simple attention/vigilance and that this effect would be independent of changes in BPR and HRR.

## Materials and Methods

### Participants, sample size and ethical approval

The protocol for this trial and CONSORT checklist are available under S1 Checklist and S1 Protocol. Twenty eight female and twenty male undergraduate volunteers (mean age 20.2 years, S.D. 2.4) were recruited in the study that was approved by the ethics committee of Sunway University Department of Psychology and complied with the Declaration of Helsinki. The study is registered in ClinicTrials.gov (identifier: NCT02386852). Sample size was determined using G*Power to establish a power level of 80% based on repeated measures ANOVA analyses with an estimated partial eta squared of 0.19. Additionally, the current sample size is equivalent to several published clinical trials utilizing a crossover design investigating, for example, the effect of ginseng on glycemic outcomes [53–56]. These trials were assessed by the Heyland Methodological Quality Score (MQS) and achieved an average score of 8.6 where a score ≥ 8 represents a trial of high quality. Participants were excluded from the study based on a number of criteria. Exclusion criteria included: those that regularly consume caffeine, Panax Ginseng or Ginkgo Biloba, those that are diabetic or have hormone sensitive conditions, autoimmune diseases, bleeding conditions, heart conditions or take medications that are known to interact with either Panax Ginseng or Ginkgo Biloba. After screening and prior to participation, each volunteer signed an informed consent form.

### Cognitive measures

Cognitive testing was carried out using the Psychology Experiment Building Language (PEBL) test battery [57]. This is a free open source software written in C++ which allows researchers to design their own experiments from scratch, or modify a pre-existing battery of cognitive tests. Since 2008, over 150 articles have been published using PEBL [58]. In the present study, some of the constituent tasks of the PEBL test battery were modified to shorten the total number of trials. Presentation of tasks occurred via laptop computers using VGA colour monitors and to complete the entire set of tasks, participants took approximately 30 minutes.

#### Visual search

This task measures selective attention and working memory [59]. Participants were initially shown the identity of a target defined by shape or colour. Following this, a screen appeared in a field of distractors and participants were required to search for the target. On some trials, the target was present whereas in others it was absent. If the target were present, participants would click on the location of the target. If the target were absent, participants would click on the “None” button. Task measures included accuracy (%) and reaction time (ms).

#### PEBL Psychomotor Vigilance Task (PPVT)

This tasks measures selective attention and simple reaction time and has been used to monitor vigilance in pilots [60]. Participants were required to respond as fast as possible whenever a red circle appeared in the middle of the screen. Task measures were reaction time (ms) and number of responses that were made before the stimulus appeared or responses that were too slow (>500 ms).

#### Stroop task

This tasks measures selective attention, response inhibition (impulsivity) and cognitive flexibility. Participants were required to determine the colour that words appeared in. In some trials, the words would correspond to actual colour names. When this was the case, participants had to ignore the written colour name and instead select the colour of the word. Task measures were reaction time (ms) for congruent, incongruent and neutral stimuli and total number of errors.

#### Bechara’s gambling task

This task is an adaptation of the Iowa Gambling task. It is widely believed to measure decision making and impulsivity [61]. Participants were required to select a card from one of four decks. Each card was associated with a possible reward (money gained) or loss (money lost) from an initial starting point of $2000. The goal of this task was to maximize the amount of money gained from the selection of cards. The task measure was the total amount of money won.

#### Berg’s card sorting test

This task is an adaptation of the Wisconsin Card Sorting Test (WCST) and measures complex executive functioning such as planning, cognitive flexibility, response inhibition, numerical skills and rules induction [62]. Participants were required to categorize cards based on the pattern appearing on them. Each pile of cards had a different colour, number and shape. A sample card would appear on the screen and participants were required to match this with one of the four piles of cards depending on a rule. Task measures included total number of errors and perseverative errors.

#### Tower of London

This task is a variant of the Tower of Hanoi problem [63] and is believed to tap into overlapping cognitive domains with the WCST [64]. Participants were required to move a pile of disks from their original configuration to the one shown on top of the screen. To do this, they could move one disk at a time with the additional restriction that disks could not be moved to a pile that had no further space. Task measures included average move time and number of excess moves.

### Physiological measures

#### Blood pressure and heart rate

Blood pressure and heart rate readings were monitored using a wrist blood pressure monitor (Omron RS3, Japan). Systolic, diastolic, and heart rate readings were taken twice (averaged) at baseline (before ginseng, ginkgo or placebo administration), 60 minutes after one of the compounds was administered, and then after the completion of each block of cognitive tasks for a total of five measurements.

#### Extracts and treatments

**Fig 1:**
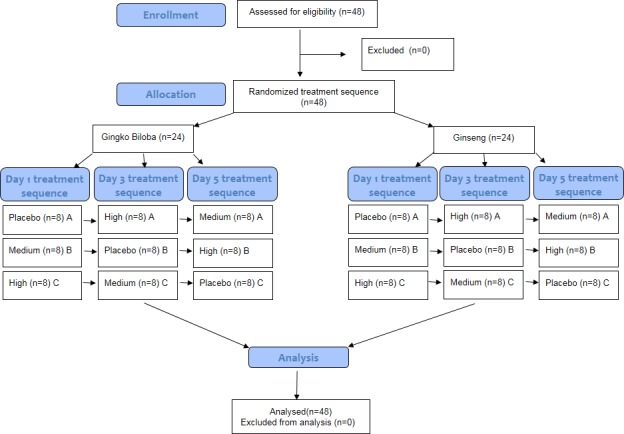
Flow diagram graphically describes the design of the study: enrolment, intervention, follow-up and data analysis. In treatment sequence, the capital letters A, B, C represent the three possible combinations of treatment sequence (group A = Placebo-High-Medium; group B = Medium-Placebo-High; group C = High-Medium-Placebo).

Half of the participants (n = 24, 16 females and 8 males) received two different doses of ginseng and a placebo over three different days, and the other half (n = 24, 12 females and 12 males) received two different doses of ginkgo biloba and a placebo. The ginkgo group received two capsules containing either 240 mg or 120 mg of ginkgo biloba extract standardized to 24% ginkgo flavone glycosides and 6% terpene lactones (GBE24/6) (MediPharm Industries, Malaysia) or similarly looking placebo capsules. The ginseng group received two capsules containing either 1000 mg or 500 mg of Panax Ginseng extract standardized to 3% of ginsenosides (GNC, USA) or similarly looking placebo capsules. Cognitive enhancing effects by ginkgo and ginseng have been reported most frequently using the above dose range. The allocation of treatment was based on the study’s Latin square, and a disinterested third party was responsible for dispensing the treatments in order to keep the procedure double-blind (see Fig 1).

### Procedure

Participants were required to attend a practice day and three testing days (ginseng/ginkgo medium dose, high dose, and placebo). Each testing day was separated by a 48 hours break to ensure a sufficient washout period between conditions [65]. Training and testing was conducted in research-dedicated laboratories. A Latin Square design was adopted to counterbalance the order of treatments over the three days of testing for all participants.

**Fig 2:**
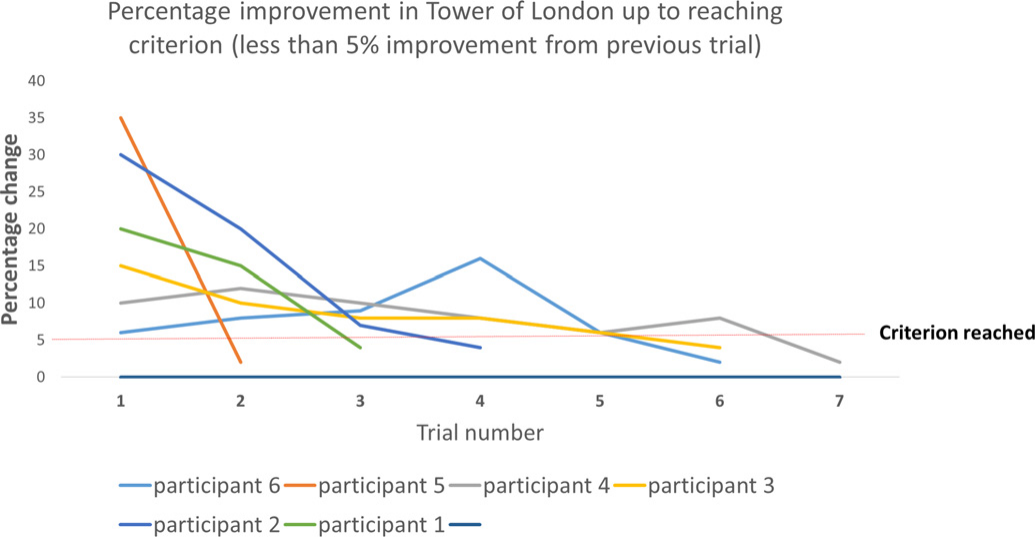
Learning rate for six participants during cognitive training in one of the six cognitive tasks (i.e. Tower of London). The graph illustrates the percentage change from one trial to the next (in this task, improvement was measured as a reduction in the number of moves). Participants had to continue practicing the task (here measured as “Trials”) until their improvement from their previous trial was equal to or less than 5% (here defined as “criterion”). Thus, while some participants only required two trials to achieve criterion, others continued for as many as seven trials. This demonstrates the importance of using a criterion which measured an individual’s relative improvement from the previous trial, rather than an absolute set score of achievement (e.g. every participant has to make fewer than 10 moves), to ensure that at time of testing (i.e. during treatment), every participant had reached their near optimal best.

During the practice day, participants were required to complete each of the six cognitive tests for as long as they continued to improve from their previous attempt by at least 5% or more. Once the improvement from their previous attempt was less than 5% for all the six cognitive tests, the practice day session was terminated. This ensured that all participants had reached a level of practice that was standardized, and enabled the isolation of the effects of ginseng/ginkgo during performance rather than during the learning phase of the cognitive tasks. For a demonstration of how the present research design allowed to separate the effects of treatment on performance rather than learning, see Fig 2. This distinction, between learning and performance appears to be often ignored in the literature [66].

On the three testing days, the first step was to take participants’ blood pressure (systolic/diastolic) and heart rate readings. This was followed by ingestion of treatment. After 60 minutes, a second blood pressure/heart rate measurement was carried out. Immediately after this, cognitive testing began. A Latin Square design was also adopted to counterbalance the order of cognitive task presented over the three days of testing. This was to minimize the effect of time on cardiovascular readings. The six cognitive tasks were split into blocks of two based on the cognitive domain assessed (Block 1: Visual search and PPVT. Block 2: Stroop task and Bechara’s gambling task. Block 3: Berg’s card sorting task and Tower of London). After completing each block of tasks, blood pressure and heart rate were taken (with a three minute break in between blocks), for a total of five cardiovascular readings.

### Statistics

Statistical analyses were carried out using SPSS version 21 (SPSS Inc). Cognitive testing, blood pressure (systolic and diastolic), and heart rate readings were analysed using one-way repeated measures analysis of variance (ANOVA) for the ginseng group, and using a mixed-design ANOVA for the ginkgo biloba as we had an equal number of males and females and gender was taken as the between subjects factor. The Greenhouse-Geiser correction was applied when necessary to correct for violations of the sphericity assumption. If a main effect was found, we run planned comparisons between placebo and medium dose and between placebo and high dose whereas we used post-hoc comparisons for interaction effects in the mixed-design ANOVA with Bonferroni correction applied to limit familywise error. A *P* value of <0.05 was considered significant.

## Results

### Cognitive performance

To investigate the effects of ginseng on cognitive performance a one-way repeated measures ANOVAs was used (three levels; placebo, medium dose and high dose). In the ginkgo biloba group, because we had an equal number of males and females in the ginkgo group, data was analysed by a 2X3 mixed-design ANOVA with gender as the between-subject factor (female-male) and dose as the within-subject factor (placebo, medium and high dose). Table 1 summarises data with respect to mean, standard deviation, f-value, p-value, and effect size for the six cognitive tasks (visual search, vigilance task, Stroop, Iowa, Berg and Tower of London) over a total of eight measures. For the Stroop task, reaction time and number of errors are reported, and for Berg task, the total number of errors and perseverative errors. The remaining four cognitive tasks had only one behavioural measure analysed. The table also includes a pre-dose measure of cognitive performance (labelled “Baseline”), which corresponds to the cognitive score obtained by each participants in the last training session (i.e. also an individual’s best score in training). In separate analyses (see S1 Results), we compared these values with those obtained during placebo. Paired sample t-tests confirmed that there were no differences between measures of cognitive performance pre-dose (i.e. during the last session of training) with cognitive scores after placebo. These findings highlight that cognitive training pre-dose had established near maximal cognitive scores, and that any improvement due to treatment was likely a performance effect rather than one due to learning. We here further report only the significant effects either by dose or dose*gender interaction (but not by gender alone) (see Table 1 for the full data set). There was a significant interaction effect by dose*gender [*F* (2, 44) = 4.98, *p* = 0.011, *η*^2^ = 0.19] of ginkgo biloba with respect to total errors in the Berg task. Post-hoc comparisons showed that in females, the medium and high dose reduced the number of Berg errors compared to placebo (*p =* 0.039 and *p =* 0.013 respectively). There was also a significant interaction effect by dose*gender [*F* (2, 44) = 6.40, *p* = 0.004, *η*^2^ = 0.23] of ginkgo biloba with respect to the number of perseverative errors in the Berg task. Post-hoc comparisons showed that in females the high dose reduced the number of perseverative errors (*p* = 0.020) but increased perseverative errors in males (*p =* 0.014). Finally, in the Stroop task there was a significant interaction effect by dose*gender [*F* (2, 44) = 4.47, *p* = 0.017, *η*^2^ = 0.17] of ginkgo biloba with respect to the number of errors in the Stroop task. Post-hoc comparisons showed that in females both the medium and high dose reduced Stroop errors (*p* = 0.027 and *p* = 0.047 respectively). There were no additional effects in any of the other cognitive tasks/measures. In the ginseng group, neither the medium nor the high dose had any effect on any of the cognitive tasks/measures.

**Table 1:** Effects of Ginseng and Ginkgo Biloba on cognitive performance.

	Baseline M SD	Placebo M SD SD	Medium M SDSD SD	High M SD	F	P	$\eta_{p}^{2}$ *η* 2	Planned comp/post
**Condition**		**Visual Search Reaction Time**						
Ginseng	859±179	853±188	889±z62	907±296	(2,46) = 1.59	0.215	.065	
Ginkgo	1039±307	1064±340	1114±201	1057±212	(2,44) = 1.13	0.332	.049	
		**Perceptual Vigilance Task Reaction Time**						
Ginseng	354±100	364±127	325±51	325±48	(1.1,26.3) = 3.0	0.086	.118	
Ginkgo	445±183	440±180	482±202	436±115	(1.4,32.7) = 1.27	0.290	.055	
		**Stroop Reaction Time**						
Ginseng Ginkgo AllGinkgo FGinkgo M	701±131	698±132	693±143	693±131	(2,46) = 0.97	0.908	.004	
Ginkgo	717±94	732±110	742±100	736±123	(2,44) = 0.19	0.822	.009	
		**Stroop Total Errors**						
Ginseng Ginkgo	3.79±2.7	3.62±2.5	4.66±2.8	4.62±3.1	(2,46) = 1.98	0.149	.079	
Ginkgo (all)	7.70±6.9	7.62±5.9	7.20±7.0	6.83±4.3	(2,44) = 0.24	0.787	.011	
Ginkgo F	7.41±9.1	6.83±7.7	3.08±3.8	3.75±2.3	(2,44) = 4.47 (2,44) = 4.47	0.017*	.169	p<m*,h*
Ginkgo M	8.00±4.1	8.41±3.5	11.33±7.1	9.91±3.6				
		**Iowa Total Points**						
Ginseng	3538±1282	3490±1234	3651±1333	3668±1404	(2,46) = 0.47	0.627	.020	
Ginkgo	2993±1416	2928±1232	2451±1192	2825±1336	(2,44) = 1.28	0.287	.055	
		**Berg Total Errors**						
Ginseng	20.9±8.2	20.4±7.8	20.2±7.6	18.8±6.8	(2,46) = 0.72	0.489	.031	
	20.3±5.8	19.6±5.7	19.0±5.4	18.7±4.4	(2,44) = 0.35	0.705	.016	
Ginkgo F	22.4±6.5	21.5±5.0	17.4±4.9	18.0±4.6	(2,44) = 4.98 (2,44) = 4.98	0.011* 0.011*	0.185 0.185	p<m*,h*
Ginkgo M	18.3±4.5	17.8±5.9	20.5±5.6	19.5±4.2				
		**Berg Perseverative Errors**						
Ginseng	14.7±5.7	14.0±5.1	13.8±5.6	13.3±6.5	(2,46) = 0.20	0.819	.009	
Ginkgo (all)	12.9±3.7	13.2±3.6	13.0±3.2	13.3±3.9	(2,44) = 0.63	0.939	.003	
Ginkgo F	14.8±4.0	14.5±3.9	12.5±3.7	11.5±3.5	(2,44) = 6.40 (2,44) = 6.40	0.004**0.004**	.226 .226	p<h*
Ginkgo M	11.1±2.5	12.0±2.9	13.5±2.7	15.0±3.6	(2,44) = 6.40	0.004**	.226	p<h*
		**Tower Of London Excess Moves**						
Ginseng	44.6±21.5	44.1±23.3	47.3±22.5	47.3±20.4	(2,46) = 0.41	0.665	.018	
Ginkgo	51.6±30.9	48.7±33.6	45.7±36.1	41.0±23.2	(2,44) = 0.71	0.494	.005	

Notes. Ginkgo F and M refer to female and male participants.

*indicates significance at *P*<0.05 and

** at the <0.01 level. Data for females and males is reported only for those measures in which there was a significant dose*gender interaction.

In all other cases, only the main effects are displayed. In the planned comparison/posthoc tests, p = placebo, m = medium dose, h = high dose.

### Cardiovascular response: pre-treatment (time 1) and after treatment (baseline or time 2)

**Fig 3:**
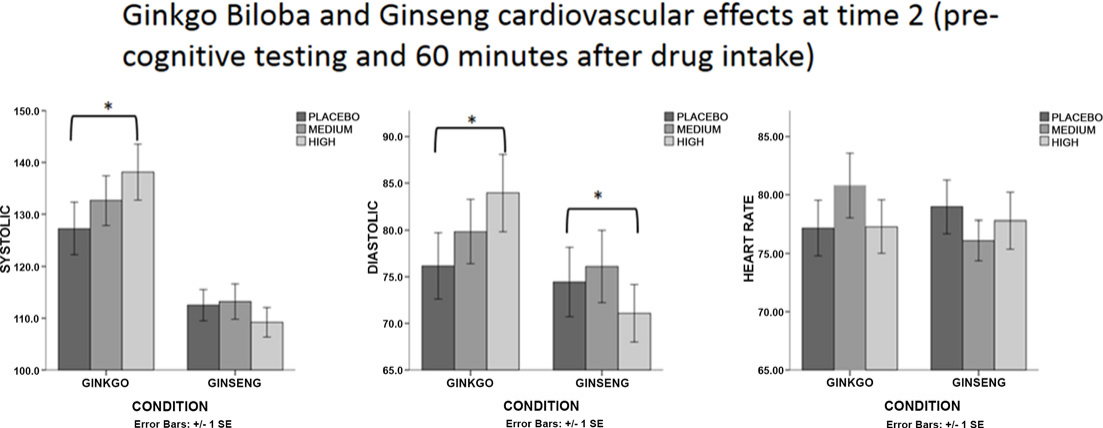
Cardiovascular data with respect to systolic, diastolic and heart rate readings for ginkgo and ginseng sixty minutes after treatment intake (also referred to as “time 2”) and before cognitive testing. Comparisons are made for placebo, medium and high dose.

We wanted to understand whether there were differences in cardiovascular activity between treatments (placebo, medium, high dose; ginseng/ginkgo) prior to drug administration (i.e. time 1). There were no main or interaction effects in the ginkgo biloba or in the ginseng group with respect to systolic, diastolic or heart rate readings. Additional analyses were then carried out to investigate whether cardiovascular changes occurred sixty minutes after ginseng/ginkgo treatment (time 2) between different doses (Fig 3). In the ginkgo biloba group, there was a main effect of dose on systolic readings [*F* (2, 44) = 3.44, *p* = 0.041, *η*^2^ = 0.14] and diastolic readings [*F* (2, 44) = 3.40, *p* = 0.042, *η*^2^ = 0.13]. Planned comparisons showed that systolic and diastolic readings were significantly higher in the high dose condition than in the placebo [*F* (1, 22) = 4.91, *p =* 0.037, *η*^2^ = 0.18] [*F* (1, 22) = 5.98, *p* = 0.023, *η*^2^ = .0.21] respectively. There was no significant interaction effect by dose*gender in terms of both systolic and diastolic readings, although there was a medium effect size for diastolic blood pressure (η² = 0.12) and a main effect of gender [*F* (1, 22) = 5.47, *p* = 0.029, η² = 0.19]. In the ginseng group, there were a main effect of dose on diastolic readings [*F* (2, 46) = 6.07, *p* = 0.005, η² = 0.21]. Planned comparisons revealed that the high dose significantly decreased diastolic readings compared to placebo [*F* (1, 23) = 6.07, *p =* 0.047, *η*^2^ = 0.16].

### Cardiovascular response to cognitive task: between treatment comparison

**Fig 4:**
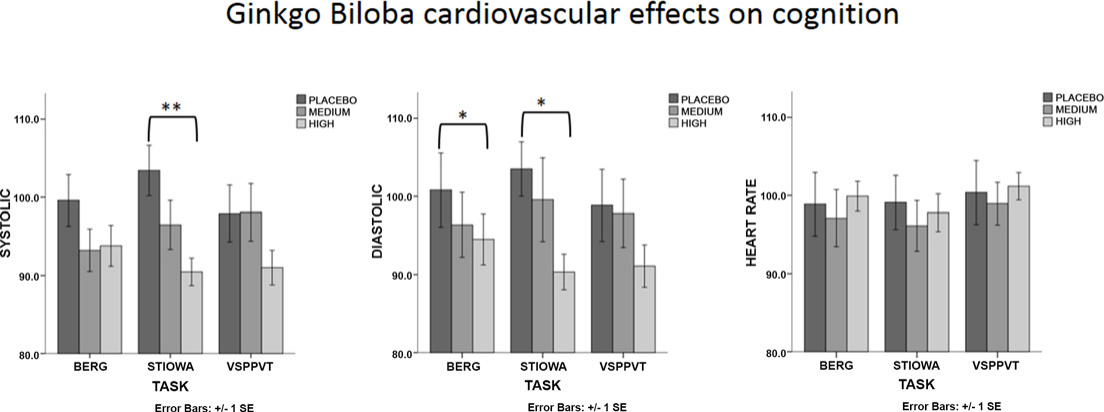
Cardiovascular data results with respect to systolic, diastolic and heart rate readings for participants in the Ginkgo Biloba group after completion of the cognitive tasks Berg, Stiowa and Vsppvt. Comparisons are between placebo, medium and high dose. Cardiovascular readings are expressed with respect to response differences between cognitive task and baseline, that is, 60 minutes after drug treatment and prior to cognitive testing (where an equal cardiovascular response to baseline = 100). * indicates statistical significance at the *p* <0.05 level.

To understand the impact of treatment (ginseng and ginkgo) on cardiovascular reactivity during cognitive testing, systolic, diastolic and heart rate raw readings were transformed into percentages in relation to baseline (time 2). For example, a systolic reading of 135 measured after completing the BERG task during a high dose of ginseng was divided by a systolic reading of 130 at baseline (time 2, before cognitive testing and 60 minutes after treatment) and reported as 103.8% of the baseline. These normalised scores were then compared across dose (placebo, medium and high) and cognitive task (BERG, STIOWA and VSPPVT). The reason for transforming these data was that treatment (both ginseng and ginkgo) had on effect on cardiovascular readings at time 2 (i.e. prior to cognitive testing). Thus, to capture the effect of treatment on cognitive testing, a relative measure of cardiovascular change was taken with respect to baseline so that each level of treatment could be meaningfully compared with one another. In fact, data comparison using raw readings would have failed to detect any effect of treatment on cardiovascular readings during cognitive testing (see Table 2 for the raw data). In the ginkgo biloba group (Fig 4), there was a main effect of dose on systolic and diastolic readings after STIOWA [*F* (2, 44) = 6.22, *p* = 0.004, *η*^2^ = 0.22] [*F* (2, 44) = 3.34, *p* = 0.044, η^2^ = 0.13] respectively. Planned comparisons revealed that the high dose significantly reduced systolic and diastolic readings compared to placebo [*F* (1, 22) = 12.33, *p* = 0.002, *η*^2^ = 0.36] [*F* (1, 22) = 10.88, *p* = 0.003, *η*^2^ = 0.33] respectively. There was a significant interaction effect by dose*gender [*F* (2, 44) = 8.89, *p* = 0.007, *η*^2^ = 0.28] of ginkgo biloba after BERG in diastolic readings. Post-hoc comparisons showed that in females the high dose significantly reduced diastolic readings compared to placebo (*p =* 0.005). There were no additional effects. Overall, effect sizes were largest for STIOWA, followed by BERG and VSPPVT.

**Table 2:** Effects of Ginseng and Ginkgo Biloba on cardiovascular reactivity.

*Physiological measure*	*Placebo (G) M SD*	*Medium (G) M SD*	*High (G) M SD*	*Placebo (GB) M SD*	*Medium (GB) M SD*	*High (GB) M SD*
**Time 1 (before treatment)**						
Systolic (ALL)	114.6±18.4	113.2±15.5	114.8±14.3	127.6±22.6	131.4±22.1	124.2±19.2
Systolic (F)				131.9±29.2	139.5±28.1	125.5±24.8
Systolic (M)				123.3±11.9	123.3±9.3	122.9±12.3
Diastolic (ALL)	74.0±16.1	74.7±14.7	72.9±13.3	78.7±18.3	78.9±16.6	74.7±12.1
Diastolic (F)				84.5±23.8	87.6±19.7	77.9±15.8
Diastolic (M)				73.0±7.9	70.2±4.7	71.5±6.1
Heart rate(ALL)	79.0±10.7	80.0±10.6	79.9±8.9	79.6±10.9	79.5±10.1	78.1±9.8
**Time 2 (after treatment, before cognitive testing)**						
Systolic (ALL)	112.5±14.7	113.2±16.7	109.2±13.9	127.2±24.6	132.6±23.2	138.1±26.5
Systolic (F)				130.3±33.5	138.9±31.4	150.2±31.8
Systolic (M)				124.2±10.9	126.4±7.5	126.0±11.4
Diastolic (ALL)	74.4±18.2	76.1±19.0	71.0±15.1	76.1±17.4	79.8±16.7	83.9±20.1
Diastolic (F)				80.0±23.1	86.0±21.2	94.8±22.6
Diastolic (M)				72.3±8.2	73.6±7.3	73.0±8.9
Heart rate(ALL)	78.9±11.2	76.1±8.4	77.7±11.8	77.1±11.5	80.7±13.6	77.2±11.1
**After cognitive testing (Task = Visual search+ vigilance task)**						
Systolic (ALL)	108.4±14.7	113.1±14.8	109.2±12.0	121.8±18.0	129.1±30.0	123.8±16.7
Systolic (F)				124.5±24.5	137.0±40.5	130±20.2
Systolic (M)				119.1±7.8	121.1±10.3	117.6±9.8
Diastolic (ALL)	65.7±9.6	69.1±9.8	70.6±13.4	73.0±14.1	77.1±21.8	74.7±12.1
Diastolic (F)				76.5±16.8	85.8±27.7	79.9±14.5
Diastolic (M)				69.5±10.5	68.5±8.0	69.5±6.1
Heart rate(ALL)	73.0±9.9	74.5±8.0	78.1±14.4	75.8±8.7	79.0±11.4	77.9±10.9
**After cognitive testing (Task = Stroop+ Iowa)**						
Systolic (ALL)	112.6±16.1	110.2±14.1	107.4±13.6	129.8±23.5	126.8±26.1	123.9±20.6
Systolic (F)				133.8±32.2	137.2±33.5	131.4±25.6
Systolic (M)				125.9±9.1	116.4±7.9	116.4±10.3
Diastolic (ALL)	73.2±15.7	70.3±15.2	72.2±15.1	77.6±17.8	78.3±23.6	74.5±13.5
Diastolic (F)				84.1±22.8	89.9±28.8	80.5±15.5
Diastolic (M)				71.1±7.2	66.7±6.8	68.5±8.2
Heart rate(ALL)	75.2±11.5	75.6±14.1	77.1±11.7	75.2±9.6	76.7±13.0	75.2±11.8
**After cognitive testing (Task = Berg+ Tower of London)**						
Systolic (ALL)	111.7±16.2	111.8±17.6	119.0±11.3	124.6±20.3	122.2±22.0	128.7±26.2
Systolic (F)				130.4±25.6	126.9±30.5	137.1±33.8
Systolic (M)				118.8±11.5	117.5±5.4	120.2±11.8
Diastolic (ALL)	71.7±15.7	73.6±18.6	71.7±14.2	75.2±19.1	75.0±15.6	78.3±19.1
Diastolic (F)				84.7±22.6	79.3±19.9	85.6±24.1
Diastolic (M)				65.6±7.7	70.8±8.6	71.0±8.5
Heart rate(ALL)	75.6±10.7	74.8±11.1	77.8±12.1	75.0±10.7	77.4±13.4	76.7±9.7

Notes. Descriptive raw data of cardiovascular responses by treatment and time (time 1 and 2) plus condition (i.e. block of cognitive task). (G) = ginseng and (GB) = ginkgo biloba. (All) = both female and male. (F) = female, (M) = male. Medium and High refer to doses.

Interestingly, a comparison was made between females and males in terms of an aggregate cardiovascular score (a score based on systolic, diastolic and heart rate across cognitive tasks) during placebo, as it appeared at first glance that females had an above baseline (i.e. above 100) response in all except one measure (systolic reading after VSPPVT) and males displayed the opposite pattern, a below baseline response (below 100) in all except one measure (systolic reading after STIOWA). An independent sample t-test revealed that females had a statistically significantly higher cardiovascular response to cognitive testing during placebo (108.3 ±15.4) than males (96.5 ±4.5) *t* (20) = 2.52, *p* = 0.042, *d* = 1.08 (S1 Fig, bottom row).

**Fig 5:**
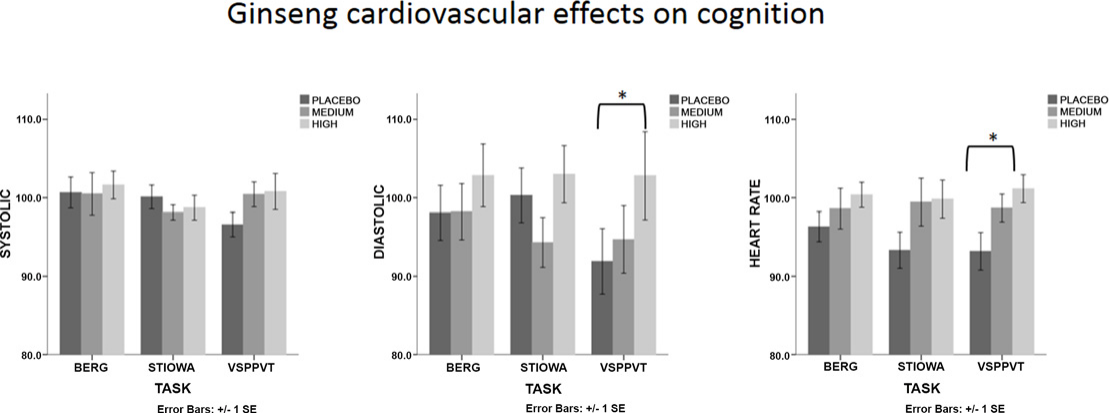
As for Fig 4, but data results are for participants in the ginseng group.

In the ginseng group (Fig 5), there was a main effect after STIOWA [*F* (2, 46) = 3.44, *p* = 0.040, η^2^ = .130]. Planned comparisons, however, revealed no significant differences between placebo and medium dose or placebo and high dose. There was also a main effect of treatment on diastolic readings after VSPPVT [*F* (2, 46) = 5.93, *p* = 0.005, η^2^ = .205]. Planned comparisons revealed that the high dose significantly increased diastolic readings compared to placebo (*p* = 0.007). Finally, there was a main effect of treatment on heart rate after VSPPVT [*F* (2, 44) = 3.79, *p* = 0.030, η^2^ = .147]. Planned comparisons revealed that the high dose significantly increased heart rate readings compared to placebo (*p* = 0.011).

### Additional analyses

The impact of cognitive task (in the absence of treatment, and in the presence of treatment with baseline as a covariate) and time (i.e. irrespective of cognitive task) on cardiovascular readings can be found in the supporting information files (S1 Results and S1 Fig).

## Discussion

The primary aim of this study was to understand whether the effects of ginseng and ginkgo biloba on cognitive performance may be associated with changes in cardiovascular reactivity. Consistent with one of the study’s hypotheses, the cognitive tasks employed in this investigation, produced differential changes in cardiovascular reactivity. More specifically, across all participants in the placebo condition, there was a significant increase in systolic blood pressure after the Stroop/Iowa block of tasks than after the Visual search/vigilance tasks, although diastolic and heart rate readings did not differ (S1 Fig, top row). This effect may reflect cardiovascular reactivity to task difficulty, as previously reported [67]. Behaviourally, there was no effect of ginseng administration (either at the medium or high dose) in any of the six cognitive tasks. Administration of ginkgo biloba in females, however, improved performance in the Stroop and Berg task whilst it impaired performance in both the Stroop and Berg task in males (in a significant manner in the case of Berg perseverative errors).

Overall, however, there was a general improvement in cognitive performance compared to placebo in six out of eight behavioural measures using either a medium or high dose of ginkgo in females but an overall worsening in performance in males (see Table 1). These findings were unexpected, and to the best of our knowledge, this is the first study to report of gender differences in cognitive performance as a result of ginkgo administration. Gender differences in psychopharmacology research have been scarcely investigated, however, there are data to suggest that these may in fact exist in particular with respect to stimulants such as caffeine and Modafinil [68, 69].

A major objective of this study, however, was to relate cognitive performance to changes in cardiovascular readings due to treatment. Data showed that the high dose of ginkgo reduced (when expressing a percentage difference between task and baseline) systolic and diastolic readings compared to placebo particularly after the Stroop/Iowa tasks, with medium-high effect sizes [70]. These significant changes can partially be explained by the fact that, during placebo, the Stroop/Iowa tasks elicited the greatest systolic/diastolic increases from baseline (i.e. above 100% of the baseline).

There was a large effect size with respect to diastolic changes also after BERG by dose*gender with the high dose of ginkgo reducing cardiovascular readings compared to placebo in females. Overall, relative decreases in systolic readings were greatest after the Stroop/Iowa tasks, and smallest after the visual search/vigilance tasks compared to placebo. This finding is of interest given that, at least in females, cardiovascular changes were specific to improved cognitive performance in the Stroop and Berg tasks but not during the vigilance and perception task. Heart rate reactivity was unaffected across all cognitive tasks (Fig 4). In the ginseng group, the high dose of ginseng significantly increased diastolic and heart rate readings compared to placebo after the visual search and vigilance tasks. This increase was present in the other tasks also but in a less pronounced manner (Fig 5). As for ginkgo biloba, these significant changes can partially be explained by the fact that, during placebo, the vigilance/perception task elicited the smallest heart rate/diastolic decrease from baseline (i.e. below 100% of the baseline). Nevertheless, and in contrast with the pattern of results observed in the ginkgo biloba group, the high dose of ginseng did not lower (i.e. below baseline) systolic, diastolic or heart rate readings across cognitive tasks. This finding is somewhat surprising, particularly given what it is known about the ability of ginseng to reduce other markers of stress [24, 25]. Moreover, some authors have argued that efficient glucoregulation may be an important mechanism by which cortisol release is diminished under a challenging cognitive test and that this reduction may in itself optimize cognitive performance [71]. Ginseng has been shown to improve glucose control and has been proposed to be a mechanism by which cognitive improvements may be modulated [72, 73]. It may be argued that considering the reduced/blunted cardiovascular response during placebo, participants in the ginseng condition may have not experienced any of the cognitive tasks as challenging, and thus ginseng may have had a limited impact in reducing physiological markers of stress (both cardiovascular and hormonal).

It is important to stress, however, that cardiovascular reactivity to a cognitive task here was measured using normalized scores. This is because both ginseng and ginkgo biloba had an unexpected but significant impact on systolic and diastolic readings one hour after intake and prior to cognitive testing. Interestingly, this effect went in opposite directions for ginseng and ginkgo (i.e. decreased diastolic readings of the high dose of ginseng compared to placebo and increased systolic and diastolic readings in the case of ginkgo). Thus, it is not the case that, for example, a high dose of gingko reduced absolute (i.e. raw) diastolic readings compared to placebo in females after completing the Berg/Tower of London tasks (see Table 2), but it is the case that there was a relative decrease in diastolic readings when computing this value measured after Berg/Tower of London against its baseline (i.e. one hour after intake and prior to cognitive testing), and expressing this as a percentage decrease from baseline. Moreover, it cannot be excluded that changes in systolic and diastolic readings at baseline may also have been unique contributors to cognitive performance. Nevertheless, it remains the case that cardiovascular changes from baseline were task specific (i.e. most evident for Stroop/Iowa and less so for the vigilance/perceptual task, see Fig 4) and time independent (S1 Results) (i.e. cardiovascular decreases from baseline were not due to the dwindling pharmacological effects of treatment as time went on but were task specific). We here further explore and discuss these and other mechanisms.

A comparison of the cardiovascular response (as a whole, see results section on how this was computed) between females and males in the ginkgo group during placebo and across cognitive tasks demonstrates that, in this sample, females responded above baseline to task whereas males displayed a blunted/reduced response to cognitive task. Moreover, this cardiovascular response was significantly different between females and males (S1 Fig, lower row). Cardiovascular reactivity in the ginseng group during placebo appeared remarkably similar to cardiovascular reactivity in the males’ ginkgo group. Interestingly, low or blunted cardiovascular reactivity to psychological stressors (e.g. Stroop, mental arithmetic tasks) has been reported to predict adverse health outcomes and personality characteristics predictive of future disease such as neuroticism, smoking, depression, obesity, alcohol abuse, exercise addiction and eating disorders [44, 74–79]. Importantly, blunted cardiovascular reactivity is also related to poor cognitive performance [40, 42, 80, 81]. This may, in part, provide an explanation for the negative or non-effects of ginseng or ginkgo in males on cognitive performance. In addition, however, the present study suggests that cognitive improvements (e.g. in the Stroop and Berg tasks) occur when a compound (e.g. ginkgo in females) is capable of reversing the original (i.e. placebo) increases in cardiovascular reactivity to a task (effect sizes were largest after Stroop and Berg and smallest after the attention/vigilance tasks). In the male group, on the other hand, although both medium and high doses of ginkgo also reversed the original cardiovascular response to a cognitive task, this effect occurred in comparison to blunted/reduced cardiovascular reactivity to a cognitive task during placebo (i.e. not above baseline). Moreover, increases (or no changes) in cardiovascular reactivity by medium or high doses of a compound (e.g. ginseng) do not appear to be associated with improved cognitive performance when blunted or reduced cardiovascular reactivity is observed during placebo (Fig 5), and neither do decreased diastolic readings at baseline (see Fig 3).

Blunted cardiovascular reactivity has been reported not only in response to stressors but also in reward-related tasks [76]. Thus, it has been suggested that similar behavioural adverse outcomes may be shared by blunted cardiovascular reactivity to stressors and rewards [82]. This is supported by evidence linking depression, obesity, addiction and ADHD to blunted cardiovascular reactivity to reward and incentive tasks [83–86]. A common neurobiological mechanism which characterises these adverse health outcomes is a dysregulation of the dopaminergic system. Nevertheless, in the current study, it is unlikely that the male participants in the ginkgo group were affected by any of the health related conditions mentioned above. A more reasonable interpretation of the data is that the blunted cardiovascular response may be caused by reduced task engagement or lower effort [79]. This could in turn result in hypoactivation of brain areas that are sensitive to psychological stress such as the amygdala and the insula and parts of the limbic system which are important for motivated behaviour [82, 87].

Additionally, the cardiovascular response may have been determined by gender differences to psychological stress. In a recent study, for example, physiological stress (i.e. cold pressor task) led males to perform better than females in a decision making task. This improvement was correlated with activations of the insula and dorsal striatum in males but not in females. Interestingly, there were neither gender difference in task performance nor in neuronal representation if participants had not been stressed [88].Other studies, however, have shown that males but not females respond with a blunted cortisol response to a psychological stress task, and that is related to increases in risk-taking behaviour and increase likelihood to relapse from smoking [89, 90]. Nevertheless, other reports looking at cardiovascular reactivity have found a blunted response to psychological stress in females but not in males [91].

Although, as mentioned previously, it is unlikely that our sample of male participants in the ginkgo group was affected by heath related outcomes which may have had an impact on cardiovascular reactivity, the baseline systolic and diastolic readings of the female participants (M = 130/80, Table 2) compared to that of males (M = 124/72) do appear unusually high. Previous research suggests that mean blood pressure in men is 6 to 10 mm Hg higher than in females in both a Caucasian and an Asian sample [92, 93]. It is possible, therefore, that ginkgo may have had beneficial cognitive effects on a selective group of slightly hypertensive individuals. It is also possible, however, that the relatively high cardiovascular baseline in females may be due to task anticipation and task engagement [79, 94]. To this end, future studies would be needed to determine how physiological correlates of task engagement may relate to cardiovascular reactivity, ginkgo administration and cognitive performance. Previous studies have used cortisol mobilization and error related negativity (ERN) as proxies for task engagement. More specifically, these studies have demonstrated that individuals who displayed higher pre-task cortisol levels, showed larger cortisol reductions soon after the cognitive task, and these measures were also related to the size of the ERN [95, 96]. Presumably, the initial cortisol increase reflects the allocation of physiological resources before cognitive testing and the decrease after completion of the task, a return to baseline. This is what was found with respect to cardiovascular reactivity in the female ginkgo group in the current study. The ERN, on the other hand, is a signal generated by the anterior cingulate cortex 60-110ms after an error response [95]. An increasing amplitude of the ERN, has been shown to be important both for task engagement but also for measures of executive functioning such as performance monitoring, response inhibition, and in predicting academic performance [95, 97–100]. This enhanced signal, may in turn have contributed to reduction in the number of errors in the Berg and Stroop tasks after ginkgo administration in females.

## Conclusions

In the current study, we aimed to understand whether specific physiological factors might help to explain ginkgo biloba and ginseng effect on cognitive performance. Our data illustrate a novel mechanism (i.e. cardiovascular reactivity) by which ginkgo biloba, but not ginseng, may be associated with certain forms of cognitive improvement, and our data show this effect to be gender dependent (i.e. specific to females). These findings provide support for a larger body of knowledge, which links stress reduction to facilitating cognitive performance. Critically, our data demonstrate that it is only in those cognitive tasks (e.g. Berg and Stroop) which elicit blood pressure increases that a compound such as ginkgo biloba may be most effective. Future studies would need to use additional biomarkers of stress that, for example, affect the hypothalamic-pituitary-adrenal (HPA) axis (e.g. cortisol) and the sympathetic nervous system (norepinephrine and epinephrine) to confirm our preliminary findings. Nevertheless, our data highlight the importance of relating drug administration not only to cognitive measures but to physiological function also. Such a methodological approach would likely benefit research on cognitive enhancement, which, over the years, has produced conflicting results.

## Supporting Information

S1 Checklist Consort 2010 checklist. (DOC)

S1 Fig Cardiovascular data results with respect to systolic, diastolic and heart rate readings for all participants in the Ginseng and Ginkgo Biloba experiments during placebo (top row). Cardiovascular measures are taken after completion of the cognitive tasks Berg (Berg+Tower of London), Stiowa (Stroop+Iowa) and Vsppvt (Visual search+PEBL Psychomotor Vigilance Task). The bottom row illustrates differences in cardiovascular response (systolic, diastolic and heart rate, averaged) to all cognitive tasks (Berg+Stiowa+Vsppvt, averaged) between females and males during placebo in the Ginkgo Biloba group. Cardiovascular readings are expressed with respect to response differences between cognitive task and baseline, that is, 60 minutes after drug treatment and prior to cognitive testing (where an equal cardiovascular response to baseline = 100). * indicates statistical significance at the *p* <0.05 level.
(TIF)

S1 Protocol Sunway University research ethics file. (DOC)

S1 Results Additional analyses. (DOCX)
