# Supplementary figures and images for: Ginseng and Ginkgo Biloba Effects on Cognition as Modulated by Cardiovascular Reactivity: A Randomised Trial
Source: PLoS One. 2016 Mar 3;11(3):e0150447. doi: 10.1371/journal.pone.0150447 (PMC4777384; doi:10.1371/journal.pone.0150447)

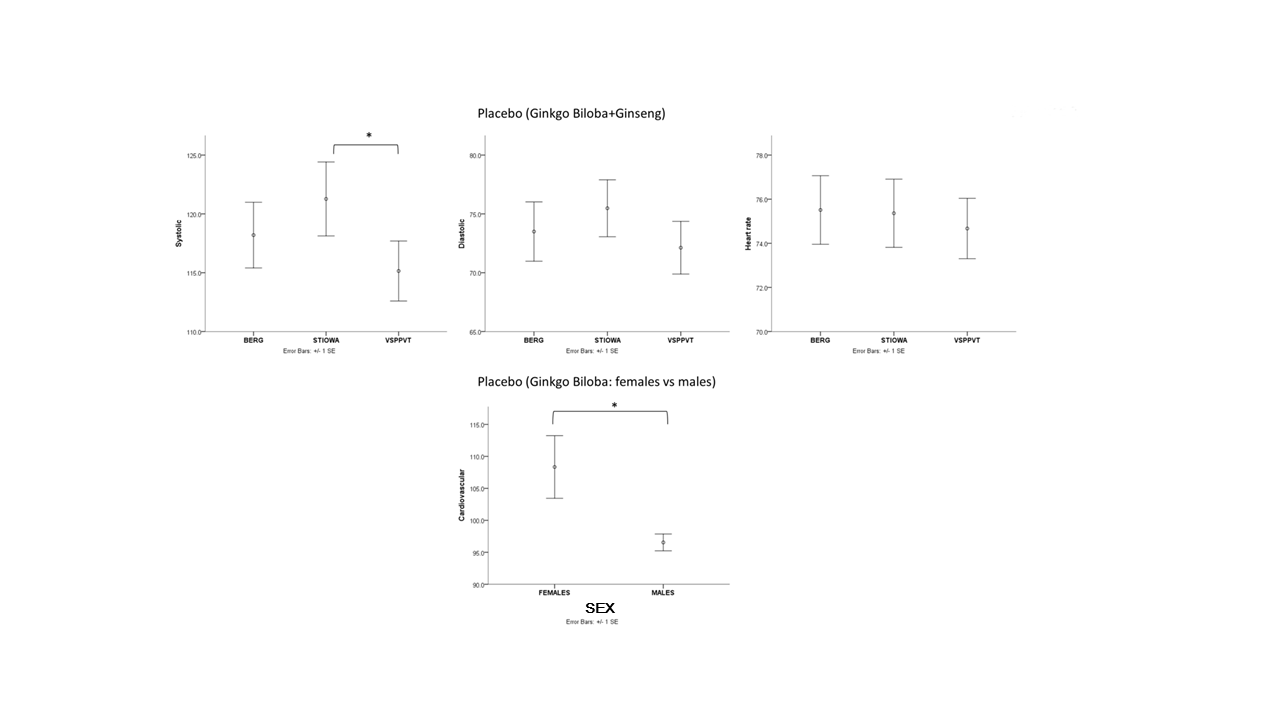

Supplement: S1 Fig — Cardiovascular measures are taken after completion of the cognitive tasks Berg (Berg+Tower of London), Stiowa (Stroop+Iowa) and Vsppvt (Visual search+PEBL Psychomotor Vigilance Task). The bottom row illustrates differences in cardiovascular response (systolic, diastolic and heart rate, averaged) to all cognitive tasks (Berg+Stiowa+Vsppvt, averaged) between females and males during placebo in the Ginkgo Biloba group. Cardiovascular readings are expressed with respect to response differences between cognitive task and baseline, that is, 60 minutes after drug treatment and prior to cognitive testing (where an equal cardiovascular response to baseline = 100). * indicates statistical significance at the p <0.05 level. (TIF) [file pone.0150447.s002.tif]
