# Supplementary material for: Ginseng and Ginkgo Biloba Effects on Cognition as Modulated by Cardiovascular Reactivity: A Randomised Trial
Source: PLoS One. 2016 Mar 3;11(3):e0150447. doi: 10.1371/journal.pone.0150447 (PMC4777384; doi:10.1371/journal.pone.0150447)
Supplement: S1 Protocol — (DOC) [file pone.0150447.s003.doc]

**
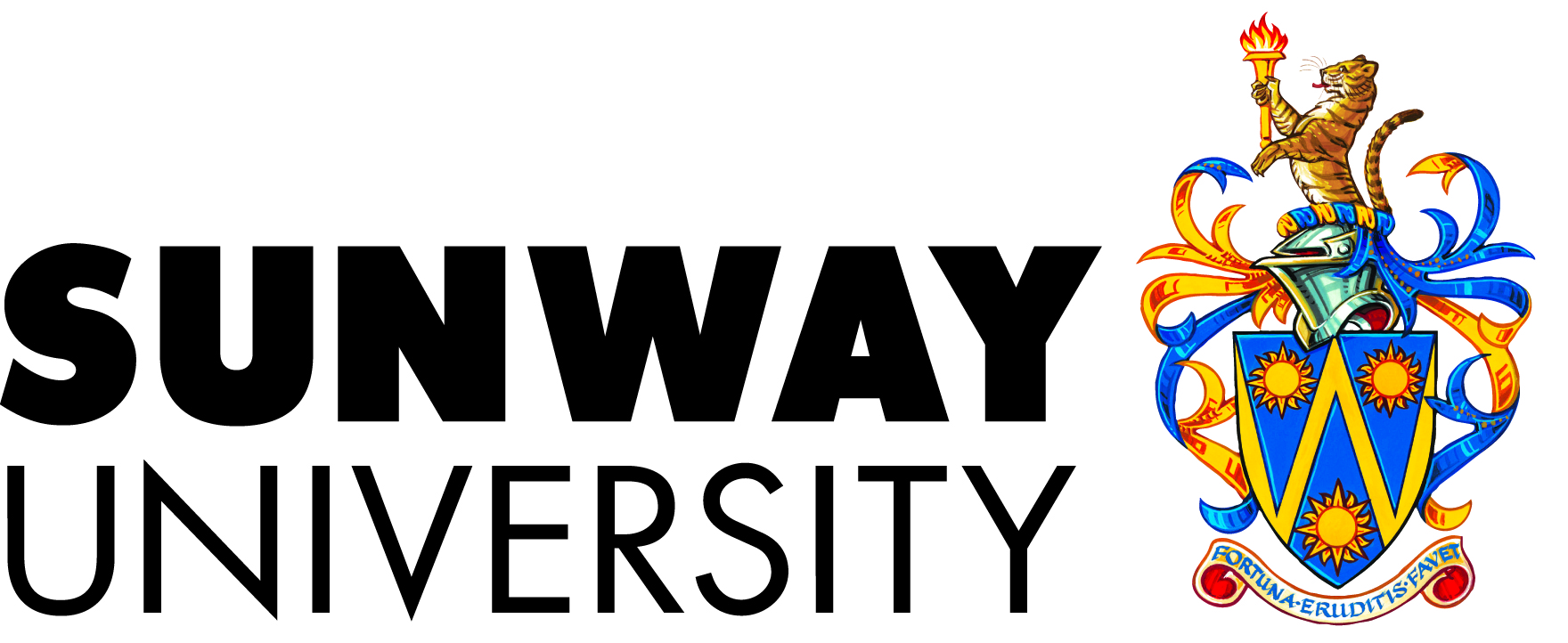
**

**Research Ethics Form**

**Ethics Committee**

**Department of Psychology**

Instructions for Researcher/s: Complete ALL sections.

SECTION 1: GENERAL INFORMATION

| 1.1 | Ethics Cycle :  (month & year) | March 2014 |  |
| --- | --- | --- | --- |
| 1.2 | Submission Type: | New Submission |  |
| 1.3 | Name of Student/s Principal Investigator: | Mariyam Muslima and Chu Jian Lim (ginkgo biloba study), Lee Xiao Shiang and Chia Mei Low (ginseng study) | |
| 1.4 | Student/ Staff ID. No: |  |  |
| 1.5 | Name of Supervisor: (for student investigators) | Dr. Luca Aquili | |
| 1.6 | Title of Research Project: | The role of Gingko Biloba and ginseng in cognitive functioning and their relationship with physiological indicators of stress | |
| 1.7 | Correspondence Address: | ______________________________________________  ______________________________________________ | |
| 1.8 | Email Address: | ___________________________ |  |
| 1.9 | Proposed duration of the study (months): | 30 weeks. |  |

SECTION 2: DETAILS OF STUDY

2.1 Please state the purpose of your research:

The purpose of the research is to study the effects of ginkgo biloba (study 1) and ginseng (study 2) on cognitive functioning and how it effects physiological indices of stress (e.g. systolic, diastolic readings and heart rate).

2.2 Please give a brief summary of research background of your study and the hypothesis of your study (word limit is maximum 1000 words, single spacing):

**Ginkgo Biloba study (Mariyam Muslima and Chu Jian Lim)**

Van Beek and Montoro (2009) and Laws,Sweetnam, and Kondel,(2010) Ginkgo biloba which is an extract from the ancient plant maidenhair tree was used in ancient china as a herbal remedy and now it is one of the most commonly used and sold herbal product around the world, mainly due to its possible beneficial effects on cognition. The active ingredients in ginkgo biloba includes flavonoids, terpenoids, ginkgolides and bilobalide (Laws, Sweetnam, and Kondel, 2010). Given its widespread use across the world, the effectiveness of this plant extract in improving cognition needs to be studied objectively.

Yoshitake, Yoshitake and Kehr (2010) did experiment with rats with chronic treatment of gingko and found an increase in dopamine and noradrenaline in the frontocortical areas of the brain. This could be the reason behind improved effects on cognition by this particular plant extract. Moreover they also suggested that ginkgo could also have an effect on enhancing mood and increasing motivation levels in people with disorders associated with monoamine oxidase inhibitors and dopamine. However they only found these effects after chronic treatment with a maintained dosage of gingko (100mg/ 14 days/ once daily) (Yoshitake,Yoshitake and Kehr, 2010). Studies done on healthy young humans such as Stough, Clarke, Lloyd and Nathan (2001) with placebo and chronic treatment of gingko for 30 (3 doses of 120mg daily) after which they were tested on a neuropsychological test battery found that treatment with ginkgo improved working memory and memory consolidation. There was no significant effect on any of the other cognitive processes tested. Majority of the researches done with ginkgo biloba which shows an improvement on cognitive functioning are with chronic treatment. Nevertheless there are researches with acute treatment which also showed some sort of change in cognitive functioning.

Elsabagh, Hartle, Osama, Williamson and File (2005) did a research with healthy university students and found that the group which was treated to acute dosage of gingko scored significantly higher than the placebo group on attention and memory task. Similarly Gertz and Kiefer (2004) did an experiment with an acute treatment of gingko 120mg and placebo and found that there was a significant improvement on secondary memory, however it had a detrimental effect on the speed of attention. Scholey and Kennedy (2002) found that treatment with single doses of gingko; all the three doses; 120, 240 and 360mgs had a positive effect on the performance of serial threes of the computerised version of the serial subtraction test compared to the placebo treatment group at 4 hour testing session. So there is some evidence that gingko does have a positive effect on cognitive functioning other than memory.

The experiments done using gingko mainly focuses on older individuals with or without cognitive impairments. Rigney, Kimber and Hindmarch (1999).did an experiment with asymptomatic healthy volunteers and found that effect of acute dosages of ginkgo is more prominent on memory than any other cognitive process. Moreover these effects were most significant with a standard 120mg dosage. They also went further explaining why the positive effects of gingko is not so visible in healthy young people as compared old people with cognitive deficits. According to them it is because healthy young people are already at the prime of their cognitive abilities and there is little room for improvement as compared to older individuals; especially those with cognitive impairments.

Like-wise Trick, and Hindmarch, (2003) in their meta-analysis said that the percentage of the improvement in cognitive functioning found were greater for older healthy individuals as compared to the younger ones. However younger participants were found to have greater improvement in objective measure when the improvement was broken down to subjective and objective. Having said that there are some inconsistencies in the literature when it comes to gingko biloba and its effects on cognitive functioning.

Snitz, O’Meara and DeKosky (2009) in the Ginkgo Evaluation of Memory (GEM) with two treatment of two daily doses of 120 mg of ginkgo biloba and placebo found that the ginkgo biloba did not result in any less cognitive decline compared to that of placebo. Correspondingly Solomon, Adams, Silver, Zimmer and DeVeaux (2002) in their study with elderly participants with healthy cognitive functioning who either received 40 mg thrice daily or similar looking placebo for 6 continuous weeks found that at the given dosage of chronic treatment ginkgo had no beneficial effects on memory and other related cognitive functions.Birks and Grimley (2009) in the meta-analysis found that the studies using ginkgo biloba in patients with cognitive impairments had inconsistent and unreliable results, thus to conclude that gingko improves or does not improve cognitive functioning would be wrong. More studies need to be done to find the correct dosage, level of improvement and also some consistency in the literature. Cognitive functioning is not the only area in which ginkgo biloba is said to have an effect on; it is also said to decrease symptoms related to acute and chronic stress.

Shah, Sharma, and Vohora (2003) found that rats who were under restraint stress which were treated to ginkgo biloba had the levels of catecholamine, serotonin and corticosterone restored to normal levels. Similarly Walesiuk, Trofimiuk, and Brazko, (2006) studied the relationship of restraint stress and cognitive functions namely memory in rats, and whether or not ginkgo helped to reduce the effect of detrimental effect of stress on memory. They found that group which was treated with ginkgo showed significantly less adverse effects on memory as compared to the rats which were unprotected. Moreover Walesiuk and Braszko (2009) found that not only did ginkgo improved the performance of rats,but it also normalised the deficits observed in rats treated with corticosterone. They said that considering the importance given in our society to perform at optimum best all the times and how stressful life is now, efficacy of herbal remedies to alleviate the effects of stress on cognition should be further studied.

**Ginseng study (Lee Xiao Shiang and Chia Mei Low)**

2.2 Please give a brief summary of research background of your study and the hypothesis of your study (word limit is maximum 1000 words, single spacing):

Ginseng is generally taken to refer to the dried root of several species in the plant genus Panax (Araliaceae family) that had been in used for thousands of years and is still one of the most used herbal medications (Ang-lee, Moss & Yuan, 2001). Two of the most recognized are Asian ginseng (*Panax ginseng C. A. Meer*) and American ginseng (*Panax Quinquefolius L*.) where small differences were found between them such as the American ginseng has a higher total of ginsenoside than Asian ginseng (Lian, Chong & Chun, 2011). Ginsenosides, are believed to be the active compounds behind the claims of ginseng’s efficacy, potential health effects and effects on the central nervous system (Christensen, 2008).

Components of ginsenosides are able to increase cell survival, extending neural growth, and rescuing neurons from death either in vivo or in vitro (Lian et al., 2011; Radad et al., 2004). A possible mechanism are the regulation of the various types of ion channel by interacting with ligand—biding sites or channel pores sites in neuronal and heterologous expressed cells (Nah et al., 2007). They are also able to inhibit voltage dependent Ca^2+^, K^+^ and Na^+^ channel activities, hence modulating neurotransmission in the brain (Liu et al., 2010, Xue et al, 2006). In addition, Petkov (1978) had found that ginseng administration can increase dopamine and norepinephrine in brainstem and serotonin in the cortex.

In animal research, intake of ginseng had been found to reduce stress and fatigue (Kennedy & Scholey, 2002). This finding was further supported by Rai, Bhatia, Sen and Palit (2003) where both acute and chronic stress in rats had been reduced, suggesting the extracts possess significant anti-stress properties. However, ginseng was also found to have a biphasic effect on stress (Kennedy & Scholey, 2002). Level of serum corticosterone in rats will be raised during cold water swim stress and ginsenoside is able to inhibit the increase of serum corticosterone but when it’s carried out on mice, it produced the opposite effect, raising level of serum corticosterone (Luo et al., 1993). In an attempt to explain the biphasic effects of ginseng, Gaffney et al. (2001) suggest that ginsenosides may inhibit catalytic enzymes resulting in increased occupancy of both negative and positive feedback stress hormone receptors and this would lead to an existing stress response in either direction being increased.

Whilst there is a good body of work attesting to the cognition-enhancing effects of ginseng with regard to animals, the evidence of such effects following chronic administration is scarce with regard to humans. In a study by D’Angelo et al. (1986), with 32 healthy young (20–24) participants, 100 mg of G115 or placebo were twice a day. After 12 weeks, tests were carried out including motor performance (finger tapping), auditory and visual simple reaction times, choice reaction times, attention (digit cancellation and digit symbol substitution), mental arithmetic performance, and logical deduction performance assessments. Within-groups analysis showed that performance in the ginseng, but not the placebo group, was significantly improved above baseline on choice reaction time, logical deduction, and cancellation tests. The second study was by Sorensen and Sonne (1996) and involved 112 healthy participants over 40 years, who was given either 400 mg of standardised ginseng extract or placebo daily for 8–9 weeks. The test results shows statistically significant performance improvements for the ginseng group, in comparison to placebo on the fastest trials of the auditory simple reaction time tests, and on the Wisconsin Card Sort Test, a putative test of ‘executive’ function. In more recent study, Kennedy et al. (2001) involved administration of 200, 400, and 600 mg of the standardised P. ginseng extract G115 and an identical placebo. The results showed that all three doses of ginseng were associated with improvements on the cognitive domain of ‘secondary memory’ through the Cognitive Drug Research computerised assessment battery. These improvements were found to be most pronounced for the middle (400 mg) dose.

In the literature reviewed, from both animal and human studies, ginsenosides, components of ginseng are able to show cognitive effects on behavioural-relevant indices. Thus, it is hypothesized that ginseng can enhance behavioural flexibility.

- 1. Please describe your study design (i.e. survey or experimental)

Experimental

- 1. Please describe the nature of your subject (i.e. number of subjects and groups, recruitment methods, inclusion and exclusion criteria of participants)

**Ginkgo Biloba study**

Twelve female and twelve male participants recruited from a Sunway university sample. The sample will exclude those that regularly consume caffeine or Ginkgo Biloba, or those that are diabetic, have experienced seizures in the past, have bleeding disorders, or take medications that are known to interact with Ginkgo Biloba. These include Ibuprofen, anticoagulants, Warfarin, Buspirone, Fluoxetine, Trazodone.

**Ginseng study**

Twelve female and twelve make participants recruited from a Sunway university sample. The sample will exclude those that regularly consume caffeine or Panax Ginseng, or those that are diabetic, hormone sensitive conditions, autoimmune diseases, bleeding conditions, heart conditions or take medications that are known to interact with Panax Ginseng. These include anticoagulants, Warfarin, Ibuprofen, MAOIs, medications that are changed by the liver, and stimulant drugs (e.g. pseudoephedrine, epinephrine).

- 1. Please describe the method of your study- detailing the procedures of the research/ treatment of participants/ compensation to participants (i.e. payments/ inducements offered), length of experiment/survey.

This will be a repeated-measure study design. Participants will be required to undertake a battery of cognitive tests designed to measure executive functioning and vigilance. Before these tests, they will be required to consume either a placebo compound, or two different doses of a Gingko Biloba extract (Ginkgo study) or ginseng (ginseng study). Blood pressure readings will also be taken before, during and after testing. The experiment will be carried out over three days, and each session will last approximately 90 minutes. Each participant will therefore be required to consume both doses of Gingko Biloba or ginseng and the placebo over the three sessions (three days) separated by a 48 hour interval to prevent carryover effects of the compounds. The experiment will be double-blind with respect to drug treatment. No compensation for taking part in the study will be offered.

- 1. Please state/ describe what materials/ instruments you will be using in the study and state whether you have obtained permission to use the copyrighted tools. Please attach a copy of your materials/ questionnaires (including the demographic questions) where relevant.

We will use the freely available software PEBL which contains the battery of cognitive tests required for this experiment. We will also be employing an easy to use wrist band blood pressure machine. Finally, a commercially available Gingko Biloba extract or ginseng extract will be administered (orally) during the experiment.

- 1. Please state how you would debrief your subjects. Please include debriefing scripts where necessary.

No deception will be involved in this experiment, as participants will be fully aware that they will be required to take one of two doses of Gingko Biloba or ginseng while completing a series of cognitive tests. Debriefing will be provided with respect to the hypotheses of the study at the end of the experiment.

- 1. For experiments, interview and study with young children, please write or attach a copy of the study introductory script/ monologue.

Not applicable.

- 1. Does your study need approval/ permission to access the study participants? If yes, please submit a copy of the permission letters to relevant organisations.

Not applicable.

SECTION 3: RISK ASSESSMENT

3.1 What particular ethical problems/ hazards do you think there are in the proposed study (i.e. dizziness, nausea, social stigma, embarrassment, loss of employment, invasion of privacy, breach of confidentiality, underage participants hazards of technical equipment, ingested compounds, discomfort or risks to participants)?

**Ginkgo Biloba study**

Gingko Biloba is considered mostly safe when used in appropriate doses (i.e. 150 mg- 600 mg, we will use a maximum dose of 240 mg), and when individuals with certain medical conditions are excluded (see answer to 2.4 and 3.2). Potential minor side effects include stomach upset, dizziness, constipation and headache.

**Ginseng study**

Panax Ginseng is considered mostly safe unless used for prolonged periods of time (e.g. >3months), and when individuals with certain medical conditions are excluded (see answer to 2.4). The most common potential minor side effect is trouble sleeping, with less common side effects including mood changes, headache and loss of appetite.

3.2 Indicate how you minimized or control the risk/ hazards.

**Ginkgo Biloba study**

We will exclude participants that may be more susceptible to the potential side effects of Gingko Biloba. These participants include diabetics, those that have experienced seizures in the past, have bleeding disorders, or take medications that are known to interact with Ginkgo Biloba. These include Ibuprofen, anticoagulants, Warfarin, Buspirone, Fluoxetine, Trazodone.

Interested participants will be informed about the possible side effects (see answer to 3.1) that may result from ingesting Gingko Biloba and will be required to sign a consent form where they declare that they understand the potential risks. In addition, every participant will be free to withdraw from the experiment at any time.

**Ginseng study**

We will exclude participants that may be more susceptible to the potential side effects of Panax Ginseng (see answer to 2.4).

Interested participants will be informed about the possible side effects (see answer to 3.1) that may result from ingesting Panax Ginseng and will be required to sign a consent form where they declare that they understand the potential risks. In addition, every participant will be free to withdraw from the experiment at any time.

3.3 How do you ensure confidentiality/ anonymity of the participants/ data (i.e. responses are kept in a lock safe, restricted access to information, no names on instruments/ questionnaires, consent form would not be stapled with questionnaires)?

Participants’ consent forms will be kept in a lock safe, and for purposes of data collection, their names will be substituted with an unrecognisable coding system (i.e. randomly generated numbers).

3.4. Have you read the British Psychological Society guidelines and principles on ethical research?

| □ Yes |  |  |  |
| --- | --- | --- | --- |

3.5 Do you agree to adhere to these principles?

| □ Yes |  |  |  |  |
| --- | --- | --- | --- | --- |

3.6 Has your supervisor/co-researcher seen and/or approved all aspects of this proposed study?

| □ Yes |  |  |
| --- | --- | --- |

SECTION 4: FUNDING INFORMATION

4.1 Is there any funding for the study?

|  |  | □ Department funding |  |  |
| --- | --- | --- | --- | --- |

Signature Date

Student/ Principal Investigator: __________________ ____________________

Supervisor/ Co-Researcher: __________________ ____________________

For Ethics Committee Use Only:

⁯□ Approved

⁯□ Approved with minor amendments

⁯□ Resubmit

⁯□ Reject

Project Ethics Number: ______________________

Signature :

Date :
