# Supplementary material for: Ginseng and Ginkgo Biloba Effects on Cognition as Modulated by Cardiovascular Reactivity: A Randomised Trial
Source: PLoS One. 2016 Mar 3;11(3):e0150447. doi: 10.1371/journal.pone.0150447 (PMC4777384; doi:10.1371/journal.pone.0150447)
Supplement: S1 Results — (DOCX) [file pone.0150447.s004.docx]

**Cardiovascular response to cognitive task during placebo**

To understand the impact of cognitive task in the absence of treatment on cardiovascular reactivity, a repeated measure ANOVA was carried out on all participants in the ginseng and ginkgo group during placebo (S1 Fig. top row). There was a main effect of cognitive task on systolic [*F* (2, 94) =3.71, *p*=0.028, η²=.073] readings. Planned comparisons revealed systolic readings taken after the Stroop and Iowa task (STIOWA) were significantly higher than readings taken after the Visual search and vigilance task (VSPPVT) (*p*=0.013). There was no main effect of cognitive task on either diastolic [*F* (2, 94) =1.42, *p*=0.245] or heart rate [*F* (1.7, 78.9) =0.26, *p*=0.736] readings.

**Cardiovascular response to time versus cognitive task**

To control for the possibility that changes to baseline were due to time, repeated measures ANOVAs were carried out between cardiovascular measurements taken after baseline irrespective of the specific cognitive task (BERG, STIOWA, or VSPPVT). If dosage had an effect on cardiovascular readings because of time, it would be expected that such readings would be higher at time 3 (closer in time to baseline) than after time 4 and 5. We specifically looked at two cardiovascular readings (systolic and diastolic readings) that were most affected at baseline by gingko administration (medium and high dose). In the ginkgo biloba group, there was no main effect of time on systolic readings at either high dose [*F* (2, 44) =0.33, *p*=0.717], or medium dose [*F* (2, 44) =0.57, *p*=0.569], and no effect of time on diastolic readings at either high dose [*F* (2, 44) =0.09, *p*=0.912] or medium dose [*F* (2, 44) =0.41, *p*=0.661]. These findings confirm that the effect of ginkgo biloba in reducing cardiovascular readings (systolic and diastolic) is task specific and time independent.

**Cardiovascular response to cognitive task: within treatment comparison**

To understand whether cardiovascular reactivity could be differentiated across block of cognitive tasks, we run a number of repeated measures ANCOVA, with baseline (time 2) acting as the covariate, and measuring at each level of treatment (i.e. placebo, medium and high dose: ginseng/ginkgo) whether systolic, diastolic, or heart rate differed between cognitive tasks (BERG, STIOWA, VSPVVT). Neither medium nor high dose of ginkgo/ginseng produced a distinct cardiovascular profile when comparing the three blocks of cognitive tasks.

S1 Table. Cognitive performance at baseline (prior to treatment) versus placebo

|  | Baseline  M SD | Placebo  M SD | Difference  M SD | *t* | *P* |
| --- | --- | --- | --- | --- | --- |
| **Condition** | **Visual search reaction time** | | | | |
| Ginseng | 859±179 | 853±188 | 5.6±61 | .436 | .667 |
| Ginkgo | 1039±307 | 1064±340 | 25.4±215 | -.577 | .570 |
|  | **Perceptual Vigilance Task Reaction Time** | | | | |
| Ginseng | 354±100 | 364±127 | 10.8±71 | -.752 | .460 |
| Ginkgo | 445±183 | 440±180 | 5.2±90 | .284 | .779 |
|  | **Stroop Reaction Time** | | | | |
| Ginseng | 701±131 | 698±132 | 3.2±52 | .302 | .765 |
| Ginkgo | 717±94 | 732±110 | 14.8±75 | -.966 | .344 |
|  | **Stroop Total Errors** | | | | |
| Ginseng | 3.79±2.7 | 3.62±2.5 | .17±1.9 | .419 | .679 |
| Ginkgo (all) | 7.70±6.9 | 7.62±5.9 | .08±4.2 | .096 | .925 |
| Ginkgo F | 7.41±9.1 | 6.83±7.7 | .58±3.2 | .615 | .551 |
| Ginkgo M | 8.00±4.1 | 8.41±3.5 | .41±5.1 | -.280 | .785 |
|  | **Iowa Total Points** | | | | |
| Ginseng | 3538±1282 | 3490±1234 | 48.1±611 | .386 | .703 |
| Ginkgo | 2993±1416 | 2928±1232 | 65.6±884 | .363 | .720 |
|  | **Berg Total Errors** | | | | |
| Ginseng | 20.9±8.2 | 20.4±7.8 | .45±7.7 | .288 | .776 |
| Ginkgo (all) | 20.3±5.8 | 19.6±5.7 | .70±2.5 | 1.346 | .192 |
| Ginkgo F | 22.4±6.5 | 21.5±5.0 | .91±2.3 | 1.349 | .204 |
| Ginkgo M | 18.3±4.5 | 17.8±5.9 | .50±2.8 | -.602 | .559 |
|  | **Berg Perseverative Errors** | | | | |
| Ginseng | 14.7±5.7 | 14.0±5.1 | .70±4.8 | .713 | .483 |
| Ginkgo (all) | 12.9±3.7 | 13.2±3.6 | .29±2.4 | -.590 | .561 |
| Ginkgo F | 14.8±4.0 | 14.5±3.9 | .33±2.0 | .573 | .578 |
| Ginkgo M | 11.1±2.5 | 12.0±2.9 | .91±2.7 | -1.171 | .266 |
|  | **Tower Of London Excess Moves** | | | | |
| Ginseng | 44.6±21.5 | 44.1±23.3 | .50±7.4 | .329 | .745 |
| Ginkgo | 51.6±30.9 | 48.7±33.6 | 2.8±12.8 | 1.100 | .283 |

Notes. Paired sample t-tests between baseline cognitive performance (prior to treatment) and cognitive

performance during placebo. *N*=24 (except for Ginkgo F and Ginkgo M where *N*=12). Difference scores were

calculated by subtracting baseline from placebo for each variable.
